# Personalized antiplatelet therapy guided by clopidogrel pharmacogenomics in acute ischemic stroke and transient ischemic attack: A prospective, randomized controlled trial

**DOI:** 10.3389/fphar.2022.931405

**Published:** 2023-01-18

**Authors:** Xiaoguang Zhang, Shanshan Jiang, Jie Xue, Ying Ding, Jingyu Gu, Liang Hu, Xushen Xu, Zhizhang Li, Yuming Kong, Youmei Li, Xiaoqiong Zhu, Yunhua Yue

**Affiliations:** Department of Neurology, Yangpu Hospital, School of Medicine, Tongji University, Shanghai, China

**Keywords:** ischemic stroke, transient ischemic attack, clopidogrel, pharmacogenomics, randomized controlled trial

## Abstract

**Background:** Clopidogrel is frequently used in patients with ischemic stroke or transient ischemic attack (TIA), but its efficacy is hampered by inter-individual variability, due to genetic differences associated with clopidogrel metabolism. We conducted this randomized controlled trial to validate whether the personalized antiplatelet therapy based on clopidogrel pharmacogenomics and clinical characteristics leads to better clinical outcomes compared with standard treatment.

**Methods:** Patients were randomly divided into the standard group or pharmacogenetic group, in which the pharmacogenetic group required the detection of the genotyping of CYP2C19*2, CYP2C19*3, and CYP2C19*17. Patients were followed up for 90 days for the primary efficacy endpoint of new stroke events, secondary efficacy endpoint of individual or composite outcomes of the new clinical vascular events, and the incidence of disability. The primary safety outcome was major bleeding.

**Results:** A total of 650 patients underwent randomization, among which 325 were in the pharmacogenomics group while 325 were in the standard group. Our study found after a 90-day follow-up, the risk of stroke and composite vascular events in the pharmacogenomics group was lower than that in the standard group. The incidence of disability significantly decreased in the pharmacogenomics group. In addition, no statistically significant differences were observed in bleeding events between the two groups.

**Conclusion:** The present study demonstrates that personalized antiplatelet therapy guided by clopidogrel pharmacogenomics and clinical characteristics can significantly improve the net clinical benefit of ischemic stroke or TIA patients during the 90-day treatment period without increasing bleeding risk.

**Clinical Trial Registration:** http://www.chictr.org, identifier ChiCTR1800019911

## Introduction

Patients with minor stroke and transient ischemic attack (TIA) carry a substantial risk of recurrent stroke and cardiovascular events (Coull et al., 2004; Amarenco et al., 2016). The Clopidogrel in High-risk patients with Acute Non-disabling Cerebrovascular Events (CHANCE) trial showed that dual antiplatelet therapy (DAPT) including aspirin and clopidogrel decreased the risk of stroke among patients with minor stroke or TIA treated within 24 h of symptom onset compared with aspirin alone (Wang et al., 2013). However, up to 8.2% of patients treated with DAPT still have recurrent stroke in the CHANCE trial. This might be related to the inter-individual variability in response to clopidogrel (Zhang et al., 2019). It is well known that hepatic cytochrome P450 (CYP) enzymes are required for the conversion of clopidogrel to its active form, but there is a 25% inefficacy of white and a 60% inefficacy of Asian patients of this conversion, while the efficacy of clopidogrel treatment is uncertain in these patients (Pan et al., 2017). Carriers of these common genetic variations in *CYP2C19* *2 and *3 alleles, which lead to a loss of functional protein and decreased clopidogrel active metabolite levels, have significantly high platelet reactivity on-treatment and a resultant increased risk of recurrent stroke and composite vascular events when treated with clopidogrel (Pan et al., 2017; Pereira et al., 2019; Xu et al., 2019).

Since genetic polymorphisms are strongly associated with clopidogrel metabolism, genotype-guided antiplatelet therapy may serve as an alternative to personalized therapy for patients with DAPT. Shen et al. (2016) divided enrolled patients into an extensive metabolizers (EM, without any *2, *3, or *17 allele) group, intermediate metabolizers (IM, with one *2 or *3 allele) group, and poor metabolizers (PM, at least two *2 or *3 alleles) group according to *CYP2C19* genotype, who received 75 mg clopidogrel daily, 150 mg clopidogrel daily, and 90 mg ticagrelor twice daily, respectively, to evaluate the clinical effects of personalized antiplatelet therapy guided by *CYP2C19* genotype and conventional DAPT in patients with acute coronary syndrome (ACS) after percutaneous coronary intervention. They found that genotype-guided antiplatelet therapy could significantly reduce the rate of major adverse cardiovascular events without increasing the risk of bleeding. However, there is limited data at present to explore the benefits of genotype-guided approach to select antiplatelet therapy for ischemic stroke and TIA patients.

It seems that ticagrelor, a direct-acting antiplatelet agent, which is independent of hepatic metabolic activation and inhibits the P2Y12 receptor on platelets (Storey et al., 2007), has a better effect on the PM patients than clopidogrel. At present, guideline in the cardiovascular field has recommended that ticagrelor is superior to clopidogrel as part of DAPT with aspirin in the treatment of patients with ACS, regardless of plans for an invasive management (Mehta et al., 2018). In the cerebrovascular field, correlation analysis in the Platelet Reactivity in Acute Non-disabling Cerebrovascular Events (PRINCE) or transient ischemic attack trial showed that patients with minor stroke or TIA carrying the *CYP2C19* loss-of-function alleles have a lower proportion of high platelet reactivity when treated with aspirin plus ticagrelor than those with aspirin plus clopidogrel (Wang et al., 2019). Therefore, for patients with *CYP2C19* loss-of-function alleles, ticagrelor may be a better choice for antiplatelet therapy to reduce cardio–cerebrovascular events. The current Ticagrelor or Clopidogrel with Aspirin in High-risk patients with Acute Non-disabling Cerebrovascular Events II (CHANCE-2) trial verified the hypothesis that DAPT including aspirin and ticagrelor would be superior to aspirin and clopidogrel in reducing the risk of subsequent strokes among Chinese patients with minor stroke or high-risk TIA carrying the *CYP2C19* loss-of-function alleles (Wang et al., 2021).

Although genotype-guided antiplatelet therapy is promising for the prevention and treatment of ischemic stroke and TIA, it should not be ignored that increasing the dose of clopidogrel or switching to ticagrelor according to the genotype may increase the risk of bleeding in clinical practices. Therefore, a fundamental balance between reducing the risk of ischemia and increasing the risk of bleeding should be carefully taken into account (Notarangelo et al., 2018) and that is why we included the clinical characteristics of individual patients into our present study.

We conducted this randomized controlled trial (RCT) to validate the hypothesis whether personalized antiplatelet therapy based on clopidogrel pharmacogenomics and clinical characteristics leads to better clinical outcomes compared with the standard treatment for patients with acute ischemic stroke or TIA.

## Materials and methods

The trial was a prospective, open-label RCT. The study protocol (ChiCTR1800019911) and data collection were approved by the hospital ethics committee (ethical approval number: LL-2018-KY-012). All patients or their representatives were provided informed consent form before the study recruitment.

### Study design and patients

From January 2019 to November 2021 in Yangpu Hospital, Tongji University School of Medicine in China, the trial enrolled patients aged over 18 years who had a mild-to-moderate acute non-cardioembolic ischemic stroke (National Institutes of Health Stroke Scale (NIHSS) score of ≤5 at the time of randomization) or those with a moderate-to-high risk of TIA (ABCD2 stroke risk score of ≥4 at the time of randomization) who could be treated with the study drug within 72 h from the symptoms onset (Zhang et al., 2019; Hou and Chen, 2020; Johnston et al., 2020). Patients were excluded from the study participation if they had intracranial hemorrhage on a baseline brain computed tomography, acute coronary syndrome, or other pathologies that could explain the neurological deficit symptoms; if their pre-mRS (modified Rankin Scale) >2; if long-term DAPT was required for >21 days; if there were contraindications to aspirin or other P2Y12 receptor antagonists; or if there was prior knowledge of the patients’ *CYP2C19* genotype.

The trial included three visits: randomization (baseline), 30 days (2 days either way), and 90 days (7 days either way). All visits involved face-to-face or telephone interviews, with data collected on electronic case report forms.

### Randomization and procedures

After the patients signed the written informed consent form, we randomly assigned the patients to the pharmacogenetic or the standard group according to a fixed-randomization schedule to ensure an approximate 1:1 ratio. A blood sample was obtained from every participant in the pharmacogenetic group immediately after randomization. Patients were categorized into five metabolite types by a *CYP2C19* metabolizer status based on *2, *3, and *17 genotypes within 24 h from admission, including ultra metabolizers (UM, *17/*17), rapid metabolizers (RM, *1/*17), normal metabolizers (NM, *1/*1), IM (*1/*2, *1/*3, *17/*2, and *17/*3), and PM (*2/*2, *2/*3, and *3/*3) (Lee et al., 2022). A pharmacogenetic group received aspirin (300 mg loading dose on day 1, then 100 mg daily until day 90) combined with a P2Y12 receptor antagonist (300 mg clopidogrel loading dose on day 1, followed by 75 mg clopidogrel once daily for UM/RM/NM, 150 mg clopidogrel once daily for IM, or 90 mg ticagrelor twice daily for PM until day 21, which can be further adjusted in combination with clinical characteristics according to a physician’s clinical experience), while the standard group received aspirin (300 mg loading dose on day 1, then, 100 mg daily until day 90) combined with clopidogrel (300 mg clopidogrel loading dose on day 1, then, 75 mg clopidogrel once daily until day 21). Clinical characteristics included age, weight, ischemic risk, history of prior stroke/TIA, bleeding risk, history of bleeding, intracranial bleeding, active bleeding, anemia, diabetes, or chronic kidney disease (Zhang et al., 2019).

### Outcomes

The primary efficacy endpoint for this trial was a new stroke (ischemic or hemorrhagic) that happened within 90 days (7 days either way). The secondary efficacy endpoint was analyzed as the individual or composite outcomes of the new clinical vascular events (ischemic stroke, hemorrhagic stroke, TIA, myocardial infarction, vascular death, or all-cause death). The other secondary endpoint of the overall disability measured as modified Rankin Scale score (mRS, score range from 0 to 6, with 0–1 indicating no disability, two to five indicating an increasing disability, and six indicating death) >1. The primary safety outcome was a major bleeding event, according to the definitions in the International Society on Thrombosis and Hemostasis (Schulman and Kearon, 2005) and Platelet-Oriented Inhibition in New TIA and Minor Ischemic Stroke trial (Johnston et al., 2013). Secondary safety outcomes included the intracranial hemorrhage and bleeding of any other causes, including minor or minimal bleeding. Definitions of the outcomes were shown in the protocol we have previously published (Zhang et al., 2019) (Supplementary Material).

### Genetic analysis

Three single-nucleotide polymorphisms for *CYP2C19*, including CYP2C19*2 (681G>A, dbSNP rs4244285), CYP2C19*3 (636G>A, dbSNP rs4986893), and CYP2C19*17 (−806C>T, dbSNP rs12248560), were genotyped in the patients assigned to the pharmacogenetic group. Multiplex allele-specific PCR was combined with a universal array developed by CapitalBio Technology Corporation, Ltd. was used to detect the *CYP2C19* loci in the human whole blood genomic DNA. Multiple amplicons of *CYP2C19* gene were amplified by multiplex PCR using allele-specific PCR primers using the human whole blood genomic DNA as templates. After amplification, the reaction mixture was hybridized with specific labeled probes immobilized on the microarray chip of the CapitalBio BioMixerTM II Microarray Hybridization Station (CapitalBio Corporation, Beijing, China). The hybridization was stopped by washing the slide with a wash buffer. The chips were scanned and imaged using a LuxScan 10K-B microarray scanner (CapitalBio Corporation, Beijing, China). Detection of polymorphic loci was obtained (Zhang et al., 2021).

### Statistical analysis

Given that, 3 months is a fairly representative timepoint for the long-term outcomes of antiplatelet therapy for stroke, and the clinical event adjudication committee recommended that the follow-up time for endpoint events be adjusted to 90 days in the analysis of the present study. Thus, the sample size recalculated on the assuming 9% cumulative incidence of the primary efficacy endpoint is in the standard group. Given an absolute risk reduction in the proportion of the primary efficacy endpoint in the pharmacogenomics group of 5%, 80% power, and a type alpha error of 5%, and the calculated sample size was 378 patients in each group.

A *t*-test was used to compare the distributions of baseline characteristics between two study groups. Proportions and χ2 test were used for categorical variables and median (interquartile range) for continuous variables. The Cox proportional hazard model was used to estimate the hazard ratios (HRs), and 95% confidence intervals associated with the primary, secondary, and safety outcomes. Also, a log-cumulative hazard plot was used to assess the proportional hazards assumption. The overall disability (the other secondary endpoint) was analyzed with the use of a logistic regression model. To estimate the cumulative incidence of endpoints, we performed the Kaplan–Meier analyses. The statistical analysis was carried out using R software (http://www.R-project.org, the R Foundation, Austria) and Empowerstats (http://www.empowerstats.com, X&Y Solutions, Inc., CA, United States). All tests were two-sided, and *p* < 0.05 was considered statistically significant.

## Results

### Demographics

**FIGURE 1:**
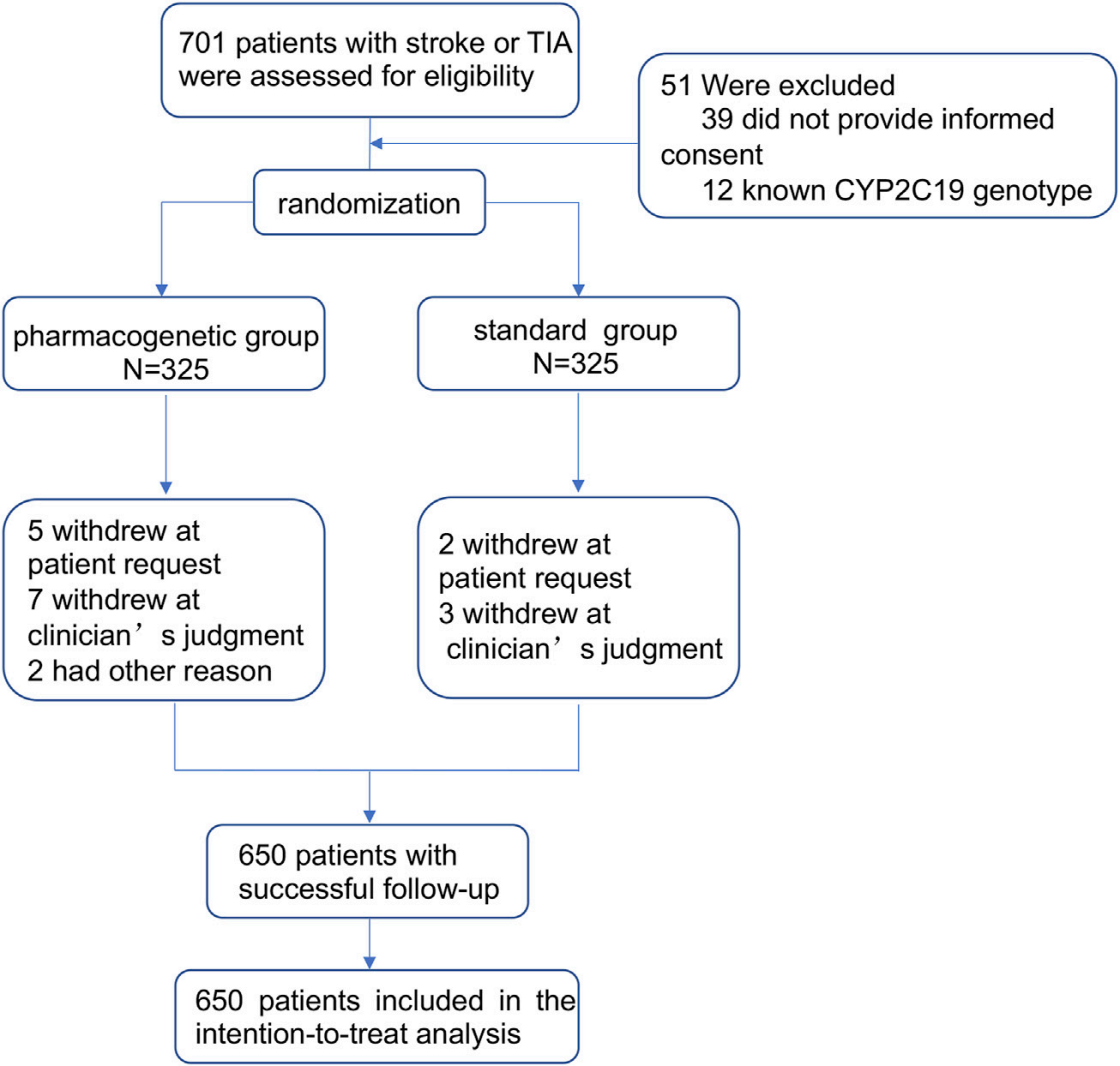
Trial profile.

Between 1 January 2019 and 30 November 2021, a total of 701 patients with stroke or TIA were assessed for eligibility, of whom 650 underwent randomization (325 in the pharmacogenomics group and 325 in the standard group). Due to disagreement or adverse events, seven withdrew at a patient’s request, ten withdrew at a clinician’s judgement, two had other reasons, and no patients were lost during the follow-up. A total of 650 patients were finally included in the intention-to-treat (ITT) analysis, as compared with the anticipated enrollment of 756 patients (Figure 1). This represents 86% of the pre-specified sample size because the data safety monitoring board opted to terminate the trial on 30 November 2021 because of the insufficient funding. The two groups were well balanced with respect to the baseline characteristics of the patients (Table 1). Most patients (89.69%) presented with ischemic stroke and 10.31% with TIA. The mean age of the patients was 67.99 years, and 73.23% of the patients were men. In total, 22 percent of patients took aspirin prior to their initial index stroke or TIA.

**TABLE 1:** Baseline characteristics of patients in the pharmacogenetic or standard group.

Characteristic	Pharmacogenetic group (*n* = 325)	Standard group (*n* = 325)
Demographic characteristics		
Age, y	67.95 ± 11.11	68.03 ± 11.28
Male, n (%)	231 (71.08)	245 (75.39)
Vascular risk factors, n (%)		
Hypertension	240 (73.85)	221 (68.00)
Diabetes mellitus	112 (34.46)	104 (32.00)
Ischemic stroke	105 (32.31)	101 (31.08)
TIA	4 (1.23)	9 (2.77)
Smoking	211 (64.92)	213 (65.54%)
BMI		
<25 kg/m^2^	187 (57.54)	221 (68.00)
25–30 kg/m^2^	121 (37.23)	87 (26.77)
>30 kg/m^2^	17 (5.23)	17 (5.23)
Clinical data		
Systolic blood pressure, mm Hg	154.90 ± 21.77	151.69 ± 21.75
Diastolic blood pressure, mm Hg	89.49 ± 13.47	87.78 ± 12.77
Baseline NIHSS, score	2 (1–3)	3 (1–4)
Pre-mRS, score		
0	284 (87.39)	288 (88.62)
1	21 (6.46)	21 (6.46)
2	20 (6.15)	16 (4.92)
Qualifying event, n (%)§		
Ischemic stroke	293 (90.15)	290 (89.23)
TIA	32 (9.85)	35 (10.77)
Drug use before randomization, n (%)		
Aspirin	82 (25.31)	64 (19.69)
Clopidogrel	14 (4.31)	18 (5.54)
Ticagrelor	0 (0)	0 (0)
Laboratory data		
Cr, mmol/L	69.00 (59.00–81.00)	69.00 (60.00–84.00)
BUN, mmol/L	5.22 (4.38–6.26)	5.17 (4.20–6.37)
Uric acid, mmol/L	324.24 ± 103.87	313.92 ± 94.09
Glucose, mmol/L	7.26 ± 2.90	7.09 ± 2.69
Triglyceride, mmol/L	1.39 (0.97–1.86)	1.36 (0.96–1.93)
Cholesterol, mmol/L	4.97 ± 1.22	4.95 ± 1.24
HDL-C, mmol/L	1.16 ± 0.28	1.15 ± 0.29
LDL-C, mmol/L	3.19 ± 0.88	3.17 ± 0.89

Values are presented as mean ± standard deviation (SD) or number (percentage) of patients as appropriate. * NA denotes not applicable. TIA, transient ischemic attack; BMI, body mass index; NIHSS, National institutes of health stroke scale; mRS, modified Rankin Scale; Cr, creatinine; BUN, blood urea nitrogen; HDL-C, high-density lipoprotein cholesterol; LDL-C, low-density lipoprotein cholesterol.

### Genotypes

The genotype distributions of patients in the pharmacogenomics group were shown in Table 2. In brief, genotyping revealed that 41.0% had at least one copy of the *CYP2C19* loss-of-function allele (*2), and 13.5% were homozygous; 8.3% had at least one copy of the *CYP2C19* loss-of-function allele (*3), and 1.0% were homozygous; and 0.9% had at least one copy of the *CYP2C19* gain-of-function allele (*17), and there was no homozygous. The proportion of *CYP2C19* metabolite types were UM/RM/NM 38.8%, IM 45.5%, and PM 15.7%, respectively. Due to the patient’s clinical characteristics, it was also taken into account in the clinical decision-making process. The actual distribution of the P2Y12 receptor antagonists used in the acute phase by the pharmacogenomics group was 75 mg clopidogrel in 66.8% of patients, 150 mg clopidogrel in 24.0%, and 180 mg ticagrelor in 9.2%.

**TABLE 2:** Frequency distribution of genetic variants and selected a P2Y12 receptor antagonist.

Characteristic	Pharmacogenetic group (*n* = 325)	Standard group (*n* = 325)
CYP2C19*2 genotype, No. (%)		
AA (*2/*2)	44 (13.5)	NA
GA (*1/*2)	133 (41.0)	NA
GG (*1/*1)	148 (45.5)	NA
CYP2C19*3 genotype, No. (%)		
AA (*3/*3)	3 (1.0)	NA
GA (*1/*3)	27 (8.3)	NA
GG (*1/*1)	295 (90.7)	NA
CYP2C19*17 genotype, No. (%)		
CC (*1/*1)	322 (99.1)	NA
CT (*1/*17)	3 (0.9)	NA
Metabolite types		
UM/RM/NM	126 (38.8)	NA
IM	148 (45.5)	NA
PM	51 (15.7)	NA
P2Y12 receptor antagonist, No. (%)		
Clopidogrel 1^a^	217 (66.8)	325
Clopidogrel 2^b^	78 (24.0)	NA
Ticagrelor	30 (9.2)	NA

* NA denotes not applicable.

^a^ Clopidogrel 1, clopidogrel 75 mg qd.

^b^ Clopidogrel 2, clopidogrel 150 mg qd.

### Outcomes

**FIGURE 2:**
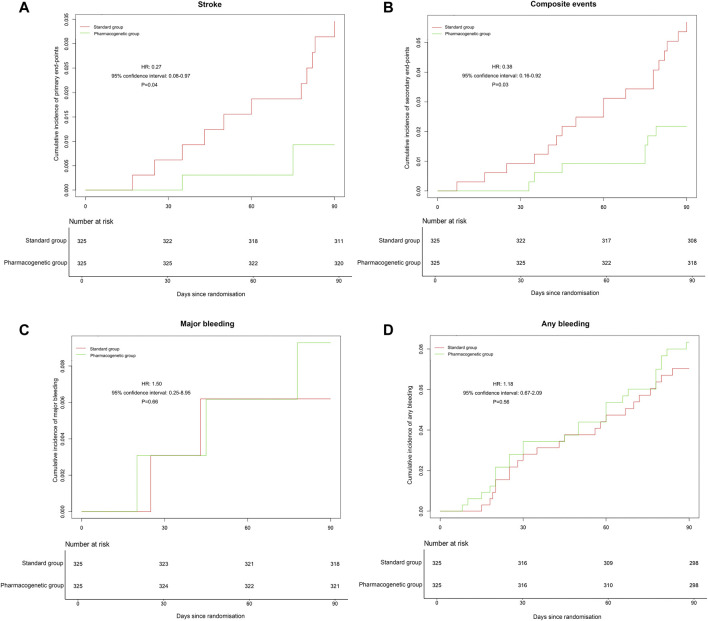
Occurrence of stroke, composite vascular, and bleeding events. **(A)** Cumulative incidence of the primary endpoint (stroke) in the pharmacogenetic or standard group at 90 days. **(B)** Cumulative incidence of the secondary endpoint (ischemic stroke, hemorrhagic stroke, TIA, myocardial infarction, vascular death, or all-cause death) in the pharmacogenetic or standard group at 90 days. **(C)** Cumulative incidence of the major bleeding in the pharmacogenetic or standard group at 90 days. **(D)** Cumulative incidence of any bleeding in the pharmacogenetic or standard group at 90 days. HR, hazard ratio.

**FIGURE 3:**
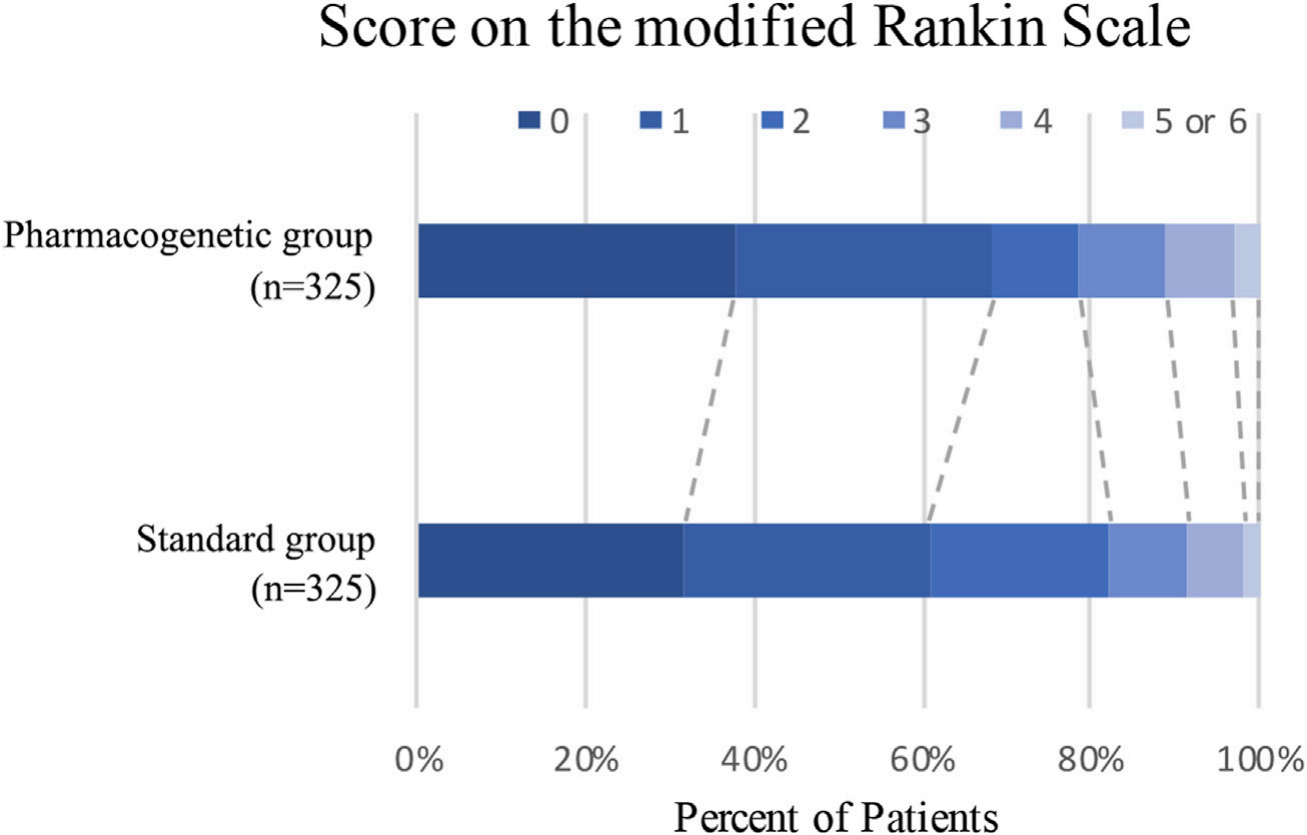
Distribution of modified Rankin Scale scores at 90 days. Distribution of scores for disability on the modified Rankin Scale among patients in the pharmacogenetic or standard group is shown. Scores vary from 0 to 6, with higher scores indicating more severe disability.

The primary efficacy endpoint occurred in three patients (0.92%) in the pharmacogenomics group and 11 patients (3.39%) in the standard group (hazard ratio 0.27; 95% confidence interval, 0.08 to 0.97; *p* = 0.04) (Figure 2; Table 3). Among these three patients with primary endpoint events in the pharmacogenomics group, two were UM/RM/NM with 75 mg clopidogrel, the other one was PM with ticagrelor. The first secondary endpoint, composite outcomes of the new clinical vascular events, occurred in seven patients (2.15%) in the pharmacogenomics group and 18 patients (5.54%) in the standard group (hazard ratio, 0.38; 95% CI, 0.16 to 0.92; *p* = 0.03) (Figure 2; Table 3). The other secondary endpoint of overall disability (mRS score >1) occurred in 30.77% of patients in the pharmacogenomics group and 40.00% in the standard group (odds ratio, 0.67; 95% CI, 0.48 to 0.92; *p* = 0.01) (Figure 3; Table 3). The primary safety endpoint (major bleeding event) occurred in three patients (0.92%) in the pharmacogenomics group and in two patients (0.62%) in the standard group (hazard ratio 1.50; 95% CI, 0.25 to 8.95; *p* = 0.66) (Table 3). An intracranial hemorrhage occurred in no patient in the pharmacogenomics group, but in 2 (0.62%) in the standard group; any bleeding events were more frequent in the patients randomized to the pharmacogenomics group: 26 (8.00%) vs. 22 (6.77%) (hazard ratio 1.18; 95% CI, 0.67 to 2.09; *p* = 0.56). Dyspnea and bleeding are common adverse events caused by ticagrelor, 5 (*n* = 30, 16.67%) patients had dyspnea, 1 (*n* = 30, 3.33%) patients had major bleeding, and 5 (*n* = 30, 16.67%) patients had minimal bleeding (Supplementary Table S1). The rate of drug discontinuation caused by dyspnea was 10.00% (3/30) and major bleeding was 3.33% (1/30) in the ticagrelor group (Supplementary Table S2).

**TABLE 3:** Effect of the pharmacogenetic group and standard group on efficacy and safety outcomes within 3 months.

Vascular event	Pharmacogenetic group (*n* = 325)	Standard group (*n* = 325)	Treatment effect (95% CI)^d^	P
Primary efficacy outcomes^a^				
Stroke, n (%)	3 (0.92)	11 (3.39)	0.27 (0.08–0.97)	0.04
Secondary efficacy outcomes				
Composite events, n (%)^b^	7 (2.15)	18 (5.54)	0.38 (0.16–0.92)	0.03
Ischemic stroke, n (%)	3 (0.92)	9 (2.77)	0.33 (0.09–1.22)	0.10
Hemorrhagic stroke, n (%)	0	2 (0.62)	N/A	N/A
Transient Ischemic Attack, n (%)	1 (0.31)	2 (0.62)	0.50 (0.05–5.49)	0.57
Myocardial infarction, n (%)	1 (0.31)	1 (0.31)	1.00 (0.06–15.94)	1.00
Vascular death, n (%)	0	2 (0.62)	N/A	N/A
Death from any cause, n (%)	2 (0.62)	6 (1.85)	0.33 (0.07–1.65)	0.18
Overall disability, n (%)	100 (30.77)	130 (40.00)	0.67 (0.48–0.92)	0.01
Primary safety outcomes^c^				
Major bleeding, n (%)	3 (0.92)	2 (0.62)	1.50 (0.25–8.95)	0.66
Secondary safety outcomes				
Intracranial hemorrhage, n (%)	0	2 (0.62)	N/A	N/A
Minor bleeding, n (%)	8 (2.46)	11 (3.38)	0.72 (0.29–1.79)	0.48
Minimal bleeding, n (%)	15 (4.62)	9 (2.77)	1.68 (0.74–3.85)	0.22
Any bleeding, n (%)	26 (8.00)	22 (6.77)	1.18 (0.67–2.09)	0.56

NA denotes not applicable.

^a^ Primary efficacy endpoint in this trial was a new stroke (ischemic or hemorrhagic).

^b^ Secondary efficacy endpoint was analyzed as the individual or composite outcomes of the new clinical vascular event (ischemic stroke, hemorrhagic stroke, TIA, myocardial infarction, vascular death or all-cause death). The other secondary endpoint of overall disability measured as modified Rankin Scale score >1.

^c^ Primary safety endpoint was a major bleeding event, secondary safety outcomes included the intracranial hemorrhage and bleeding of any other cause, including minor or minimal bleeding.

^d^ Treatment effects are reported as hazard ratio with 95% confidence intervals (CI) for all outcomes, except for the overall disability, for which the treatment effect is reported as odds ratio with the 95% CI. The widths of the confidence intervals for the endpoint outcomes were not adjusted for multiple comparisons and no definite conclusion can be drawn from these data.

## Discussion

In this prospective RCT, patients with mild-to-moderate acute non-cardioembolic ischemic stroke or high-risk TIA assigned to the pharmacogenomics group within 72 h after the symptoms onset have a lower risk of stroke and composite vascular events at 90 days than those who were assigned to the standard group. A net benefit in the overall disability rate was observed in the pharmacogenomics group, defined as mRS score greater than 1. In addition, the pharmacogenomics group was not significantly associated with more major, minor, minimal, or any bleeding in comparison with usual practices.

Clopidogrel remains the most widely used antiplatelet agent in the real world. However, multiple studies have shown an increased risk of major adverse cardiovascular events after percutaneous coronary intervention in clopidogrel-treated IM and PM patients compared with similarly treated UM, RM, or NM patients (Collet et al., 2009; Giusti et al., 2009; Mega et al., 2009). Pharmacogenetic detection has emerged in clinical practices to guide cardiovascular drug therapy over the past decade (Duarte and Cavallari, 2021). There was increasing evidence from ACS that obtaining genotype data early after percutaneous coronary intervention for patients with *CYP2C19* loss-of-function alleles, and thereby developing genotype-guided personalized antiplatelet therapeutic regimens could reduce the risk of major adverse cardiovascular events (Cavallari et al., 2018; Kheiri et al., 2019). Thus, we conducted this genotype-guided antiplatelet therapy in ischemic stroke and TIA to evaluate its clinical values. In consideration of the risks of bleeding, we also took the clinical characteristics of individual patients into account in this study.

In the PRINCE trial (Wang et al., 2019) involving Chinese patients aged 40–80 years who received 21 days of DAPT within 24 h of an acute minor ischemic stroke or TIA, the incidence of stroke (*p* = 0.2) or composite vascular events (*p* = 0.17) in the aspirin-ticagrelor group had no statistical difference with the aspirin-clopidogrel group. However, in terms of safety, the aspirin-ticagrelor group showed more minimal bleeding events (HR = 1.86, *p* = 0.003) and any bleeding events (HR = 1.65, *p* = 0.007) than the aspirin-clopidogrel group. In our study, the risk of stroke and composite vascular events was lower in the pharmacogenomics group than in the standard group. The incidence of the overall disability was also significantly decreased in the pharmacogenomics group. Also, we did not find a significantly higher incidence of bleeding events in the pharmacogenetic group. It is possibly because that only patients with a *CYP2C19* PM status received ticagrelor and they continued to take aspirin instead of ticagrelor after 21 days of DAPT in our trial. Moreover, their clinical characteristics were evaluated in the meanwhile. In addition, the different enrollment criteria among different studies might contribute to the inconsistent results.

Genotype-guided antiplatelet therapy was considered a promising alternative approach for personalized treatment of ACS and stroke. The Pharmacogenetics of Clopidogrel in Patients With Acute Coronary Syndromes (PHARMCLO) trial reported that a personalized approach to antiplatelet therapy based on genetic and clinical characteristics of patients with ACS reduced ischemic and bleeding events in comparison with the standard treatment (Notarangelo et al., 2018). The current CHANCE-2 trial demonstrated that DAPT with aspirin and ticagrelor was superior to aspirin and clopidogrel in reducing the risk of subsequent stroke in Chinese patients with minor ischemic stroke or high-risk TIA who were carriers of *CYP2C19* loss-of-function (Wang et al., 2021). They found a modestly lower risk of stroke at 3 months with ticagrelor compared with clopidogrel. It is self-evident that the findings of CHANCE-2 trial have an important value for the secondary prevention of stroke in Asian population. In the present study, we also found that personalized antiplatelet therapy could reduce the incidence of stroke and composite vascular events without increasing the risk of bleeding, which is consistent with the PHARMCLO and CHANCE-2 trials. However, our study design is still different from the CHANCE-2 trial. The differences between our study and CHANCE-2 trial are mainly reflected in the following points: first, the subjects included in our study and CHANCE-2 trial were different. Within 24 h of the symptoms onset, eligible patients (minor ischemic stroke or high-risk TIA) only carrying *CYP2C19* loss-of-function alleles were included in the CHANCE-2 trial. However, our study included patients (mild-to-moderate ischemic stroke or moderate to high-risk TIA) with whole genotypes who could be treated with the study drug within 72 h of symptoms onset. Second, in addition to genetic information, we also considered the clinical characteristics of patients in order to reduce the risk of bleeding. Third, the CHANCE-2 trial was a multicenter study with a larger number of subjects than ours. Although limited by the small number of participants, we also found a benefit of antiplatelet therapy based on genetic and clinical characteristics in acute mild-to-moderate ischemic stroke or TIA patients. Furthermore, more RCTs with structured and standardized study protocols based on a *CYP2C19* genotype are needed to allow finer tuning in this field.

Some limitations of this study need to be emphasized. First, the data we collected all come from a single center, so the sample size was insufficient, and expansion of the sample size in multi-centers is needed for further verification. Second, our study only involved the Chinese population, which might limit the generalizability of our findings. Third, prasugrel is not listed in China; otherwise, the alternative therapy of IM to prasugrel may have another surprising result. Fourth, since *CYP2C19* polymorphisms account for only part of the variability in the platelet response to clopidogrel, it is unclear whether a specific genetic polymorphism is able to influence the clinical prognosis for the individual patient. Also, the present study investigated the value of *2,*3, and *17 alleles, any other *CYP2C19* alleles that are frequent in Chinese population were not investigated. Fifth, the clopidogrel genetic metabolism profile of the control group was not performed, it is unknown whether the two groups were well-matched; further improvements in future research are needed. Finally, a placebo effect caused by the open-label design (Gupta et al., 2017), might lead to potential biases in the assessment of adverse events, drug continuation, and even the physicians’ or patients’ decisions (Wang et al., 2019).

In conclusion, the present study suggested that a personalized antiplatelet therapy for patients with mild-to-moderate ischemic stroke or high-risk TIA based on their genetic and clinical characteristics lead to better clinical outcomes (stroke and composite vascular events) in comparison with the standard treatment. The incidence of disability significantly decreased in the pharmacogenomics group. In addition, there were no statistically significant differences in bleeding events between the two groups. However, these results should be interpreted with caution due to the small number of patients and a short follow-up time in this study.

## Conclusion

The present study demonstrates that personalized antiplatelet therapy guided by clopidogrel pharmacogenomics and clinical characteristics can significantly improve the net clinical benefit of ischemic stroke or TIA patients during the 90-day treatment period without increasing bleeding risk.

## Data Availability

The original contributions presented in the study are publicly available. This data can be found here: https://www.ncbi.nlm.nih.gov. Accession Number: SUB12521502.
