# Supplementary material for: Personalized antiplatelet therapy guided by clopidogrel pharmacogenomics in acute ischemic stroke and transient ischemic attack: A prospective, randomized controlled trial
Source: Front Pharmacol. 2023 Jan 18;13:931405. doi: 10.3389/fphar.2022.931405 (PMC9889636; doi:10.3389/fphar.2022.931405)
Supplement: Supplementary file 1 [file DataSheet1.docx]

**Definitions of the outcomes**

Primary efficacy endpoint

The primary efficacy endpoint for this trial is a new stroke event (ischemic or haemorrhagic) that happens within 1 year. Ischemic stroke is defined as a sudden focal neurological dysfunction caused by vascular causes, duration ≥24 hours or neurological dysfunction due to imaging and clinical symptoms caused by bloody infarction rather than cerebral haemorrhage found by imaging examination. Haemorrhagic stroke is defined as acute extravasation of blood into the brain parenchyma or subarachnoid space with associated neurological symptoms.

Secondary efficacy endpoint

The secondary efficacy endpoint is analysed as the individual or composite outcomes of the new clinical vascular event (ischemic stroke, haemorrhagic stroke, myocardial infarction or vascular death). The definition of vascular death is adapted from the CHANCE trial. Briefly, vascular death is defined as death resulting from stroke (ischemic or haemorrhagic), systemic haemorrhage, myocardial infarction, congestive heart failure, pulmonary embolism, sudden death or arrhythmia.

Safety endpoint

Safety endpoint is a major bleeding event, according to the definitions in International Society on Thrombosis and Haemostasis and Platelet-Oriented Inhibition in New TIA and Minor Ischemic Stroke) Trial. Major haemorrhage is defined as symptomatic intracranial haemorrhage or intraocular bleeding causing loss of vision, requiring two or more units of red cells or equivalent amount of whole blood replacement, or requiring hospitalisation or prolongation of an existing hospitalisation, surgical intervention or death.

**Supplementary table S1. Adverse Events within 90 Days**

|  | Pharmacogenetic group  (n=325) | | | Standard group  (n=325) |
| --- | --- | --- | --- | --- |
| Adverse Events | Clopidogrel 1§  (n=217) | Clopidogrel 2 ¶ (n=78) | Ticagrelor  (n=30) | Clopidogrel 1§  (n=325) |
| Dyspnea, n (%) | 6 (2.76) | 2 (2.56) | 5 (16.67) | 9 (2.77) |
| Major bleeding, n (%) | 2 (0.92) | 0 (0.00) | 1 (3.33) | 2 (0.61) |
| Minor bleeding, n (%) | 3 (1.38) | 5 (6.41) | 0 (0.00) | 11 (3.38) |
| Minimal bleeding, n (%) | 8 (3.69) | 2 (2.56) | 5 (16.67) | 9 (2.77) |

§ Clopidogrel 1, clopidogrel 75mg qd; ¶ Clopidogrel 2, clopidogrel 150mg qd.

**Supplementary table S2. Adverse Events leading to Drug Discontinuation within 90 Days**

|  | Pharmacogenetic group  (n=325) | | | Standard group  (n=325) |
| --- | --- | --- | --- | --- |
| Adverse Events | Clopidogrel 1§  (n=217) | Clopidogrel 2 ¶ (n=78) | Ticagrelor  (n=30) | Clopidogrel 1§  (n=325) |
| Dyspnea, n (%) | 0 (0.00) | 0 (0.00) | 3 (10.00) | 0 (0.00) |
| Major bleeding, n (%) | 2 (0.92) | 0 (0.00) | 1 (3.33) | 2 (0.61) |
| Minor bleeding, n (%) | 0 (0.00) | 1 (1.28) | 0 (0.00) | 1 (0.31) |
| Minimal bleeding, n (%) | 0 (0.00) | 0 (0.00) | 0 (0.00) | 0 (0.00) |

§ Clopidogrel 1, clopidogrel 75mg qd; ¶ Clopidogrel 2, clopidogrel 150mg qd.
